# A Cell Model Suitable for a High-Throughput Screening of Inhibitors of the Wnt/β-Catenin Pathway

**DOI:** 10.3389/fphar.2018.01160

**Published:** 2018-10-11

**Authors:** Marina Grimaldi, Abdelhay Boulahtouf, Corinne Prévostel, Alain Thierry, Patrick Balaguer, Philippe Blache

**Affiliations:** ^1^Institut de Recherche en Cancérologie de Montpellier, Montpellier, France; ^2^INSERM, U1194, Montpellier, France; ^3^Université de Montpellier, Montpellier, France; ^4^Institut Régional du Cancer de Montpellier, Montpellier, France

**Keywords:** cell models, Wnt/β-catenin pathway, inhibitors, high-throughput screening, colorectal cancer

## Abstract

A constitutive activation of the Wnt/β-catenin pathway is an initiating event in colon carcinogenesis. We developed colon cancer cells models that highlight the non-selectivity of previously described inhibitors of the Wnt pathway and we propose our model as a suitable screening system for inhibitors of the pathway.

## Results

A constitutive activation of the Wnt/β-catenin signaling pathway is admitted as an initiating event of carcinogenesis in at least 90% of colorectal cancers (Giles et al., 2003). This constitutive activity is mostly due to mutations of the APC tumor suppressor that result in the accumulation of β-catenin in the nucleus where β-catenin interacts with TCFs transcription factors to activate the transcription of target genes like c-myc (Sansom et al., 2007). To date, very few molecules targeting the Wnt pathway have been discovered and none has been yet approved for clinical practice (Kahn, 2014). Therefore, there is a great interest in identifying new inhibitors of Wnt signaling for clinical use.

Luciferase-based reporter assays are widely used for studying gene expression at the transcriptional level. Here, we use such a system to set up a high-throughput screening assay for inhibitors of the Wnt/β-catenin signaling pathway by using DLD-1 cells stably transfected with a luciferase TCF reporter plasmid (Veeman et al., 2003). The choice of a good control was critical given that a previous work dedicated to screen new Wnt inhibitors had recently been retracted due to a non-selective inhibition of the firefly luciferase activity (Li et al., 2017). Besides, a reporter system based on mutated TCF binding sites is available, but has a very low basal luciferase activity and is rather a control for a non-specific activation of the Wnt pathway. Here, we developed a genetically modified DLD-1 cell line model expressing the firefly luciferase under the control of the E2F1 promoter, an independent promoter of the WNT pathway.

Two types of available Wnt inhibitors were used in order to validate the model: the tankyrase (TNKS) inhibitors XAV939 (Huang et al., 2009), IWR-1 (Chen et al., 2009) and WIKI4 (James et al., 2012), and the destabilizers of the TCF/β-catenin complex ICRT14 (Gonsalves et al., 2011) and PNU-74654 (Trosset et al., 2006). TNKS acts as an activator of the Wnt/β-catenin signaling by mediating poly-adenosine diphosphate (ADP) ribosylation of AXIN-1 and -2, two key components of the β-catenin destruction complex whose inhibition enhances β-catenin degradation and consequently inhibits the Wnt/β-catenin signaling (Yamada and Masuda, 2017).

**FIGURE 1:**
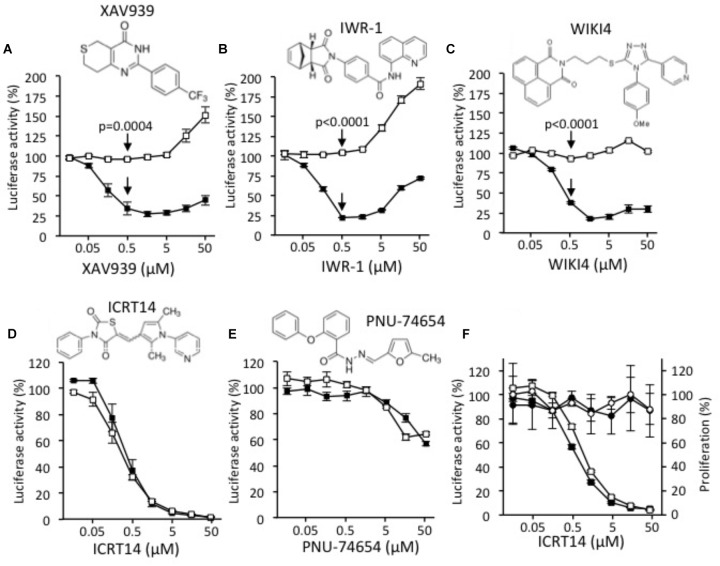
Effects of XAV939 **(A)**, IWR-1 **(B)**, WIKI4 **(C)**, ICRT14 **(D)**, and PNU-74654 **(E)** on luciferase activity of DLD1-Wnt-luc cells (black squares) and of DLD1-luc control cells (white squares). **(F)** MTT assay was performed in presence of ICRT14 on DLD1-Wnt-luc cells (black circles) and on DLD1-luc control cells (white circles). In parallel, luciferase activity of DLD1-Wnt-luc cells (black squares) and of DLD1-luc control cells (white squares) was measured. The Student’s *t*-test was performed for doses of 0.5 μM and the probability of error (*p*-value) is indicated by arrows.

XAV939 (**Figure 1A**), IWR-1 (**Figure 1B**), and WIKI4 (**Figure 1C**) specifically inhibited the activity of the Wnt/β-catenin signaling, with and IC50 of 0.13, 0.21 and 0.28 μM, respectively. However, a side activating effect was observed at doses higher than 1 μM as evidenced by the increase of the luciferase activity observed in the control conditions. Besides, both ICRT14 (**Figure 1D**) and PNU-74654 (**Figure 1E**) behaved as non-selective inhibitors as evidenced by the inhibition of both Wnt dependent and independent luciferase activities. In addition, PNU-74654 was poorly efficient. To further determine whether the apparent inhibitory effect of ICRT14 on the Wnt independent luciferase activity was due to a toxicity, or not, we evaluated the impact of ICRT14 on cells viability by using the MTT system in parallel with measurement of the luciferase activity. As shown in **Figure 1F**, ICRT14 again decreased both Wnt dependent and independent luciferase activities in a dose dependent manner but had no significant effect on cells viability.

## Materials and Methods

Luciferase and MTT assays were done as we previously described (Molina-Molina et al., 2008). More details about the methods are available in the **Supplementary Material**.

## Discussion

With respects to the use of inhibitors previously reported as specific, studies have concluded that biological activities were regulated by the Wnt/β-catenin pathway. In the present study, we demonstrate that the destabilizers of the TCF/β-catenin complex ICRT14 and PNU-74654 are unspecific inhibitors of the Wnt/β-catenin pathway. Therefore, to test the implication of the Wnt pathway in a biological mechanism, it seems more rationable to use at least one of the specific inhibitors confirmed here. Compared with the original reference system dedicated to test the impact of compounds on the activity of the Wnt/β-catenin signaling pathway, our method was set-up with an adequate control that lowers the number of false positives resulting from a non-specific inhibition of the luciferase enzymatic activity. For example, using our method points out ICRT14 as a non-specific inhibitor of the Wnt/β-catenin signaling pathway. Besides, true positives will have to be dose-dependent tested, and their ability to decrease the proliferation of colon cancer cells will have to be evaluated for further potential therapeutic purposes.

## Author Contributions

CP, PhB, and PaB designed the study. CP, PhB, and AT drafted the manuscript. MG, AB, and PhB performed the experimental work.

## Conflict of Interest Statement

The authors declare that the research was conducted in the absence of any commercial or financial relationships that could be construed as a potential conflict of interest. The reviewer ZD and handling Editor declared their shared affiliation.
