# Supplementary material for: A Cell Model Suitable for a High-Throughput Screening of Inhibitors of the Wnt/β-Catenin Pathway
Source: Front Pharmacol. 2018 Oct 11;9:1160. doi: 10.3389/fphar.2018.01160 (PMC6193374; doi:10.3389/fphar.2018.01160)
Supplement: Supplementary file 1 [file Table_1.doc]

**SUPPLEMENTARY MATERIAL**

The M50 Super 8x TOPFlash plasmid from Addgene consists of 7 TCFs binding sites (AGATCAAAGG) with a minimal TA viral promoter driving expression of the firefly luciferase gene. The neomycin resistance gene was obtained by PCR amplification from the pcDNA3-neo plasmid (Promega) and inserted in the Not1 site of M50 Super 8x TOPFlash. The EF1a-Luc2-Hyg plasmid was generated as follows. The luciferase2 sequence was obtained by HindIII and XbaI digestion of the pGL4.10 plasmid (Promega) and inserted in the pcDNA3-Hygromycin plasmid (Invitrogen). The CMV promoter of the resulting plasmid was replaced by a EF1a promoter PCR amplified from the pBudCE4.1 plasmid (Invitrogen) inserted between Bgl2 and Hhe1 restriction sites.

The DLD-1 colon cancer cells are APC-mutated and were obtained from the American Type Culture Collection (ATCC). DLD-1 cells stably expressing Super 8xTOPFlash were generated by transfection using lipofectamine 2000 (Thermo Fisher Scientific) and selected for G418 (800 µg/ml) resistance. Among the several G418 resistant clones tested for luciferase activity and the one exhibiting the higher luciferase activity was selected for the subsequent experiments. The control cell line was obtained by a stable transfection of the EF1a-Luc2-Hyg plasmid followed by a selection of hygromycin (250 µg/ml) resistant clones. The cells were handled in biosafety level 2 laboratory.

XAV939, ICRT14 and WIKI4 were from Tocris Bioscience. IWR-1 and PNU-74654 were from Sigma-Aldrich.

Luciferase and MTT assays were performed as previously described (Molina-Molina et al., 2008). Briefly, reporter cell lines (150μl) were seeded at a density of 75,000 cells per well in 96-well white culture plates with transparent bottom (Greiner CellStar). Wnt inhibitors (50μl) were added into the wells using the Biomek 3000 robot (Beckman Coulter). Cells were incubated at 37°C for 24h and culture medium was replaced with medium containing 3.10^−4^M luciferin (Promega). Luciferase activity was measured for 2s in intact living cells using a plate reader (PerkinElmer Luminometer). Tests were performed in quadruplicate for each concentration and each test was repeated at least three times.

The MTT test, was carried out after luciferase measurement by replacing the luciferin by the MTT solution (0.5 mg/ml). After 3 h incubation, viable cells cleaved the MTT tetrazolium into a dark blue formazan reaction product while dead cells remained colorless. The MTT-containing medium was replaced by DMSO and absorbance was measured at 540 nm.

EC50 and statistics were calculated using the PRISM version 5.0 GraphPad software.
